# The Role of Augmented Reality in Surgical Training: A Narrative Review

**DOI:** 10.7759/cureus.95214

**Published:** 2025-10-23

**Authors:** Mohamed Abosheisha, Raghunath Prabhu, Momen Abdelglil, Ahmed Swealem, Mohamed Ali, Zaid Al-Hamid, Rezuana Tamanna, Mahmoud Elhadidi

**Affiliations:** 1 General Surgery, Wirral University Teaching Hospital NHS Foundation Trust, Birkenhead, GBR; 2 Colorectal Surgery, Leicester Royal Infirmary, University Hospitals of Leicester NHS Trust, Leicester, GBR; 3 Pediatric Surgery, Mansoura University Children Hospital, Mansoura, EGY; 4 Orthopedics, Southmead Hospital, North Bristol NHS Foundation Trust, Bristol, GBR; 5 Surgery, Hywel Dda University Health Board, Carmarthen, GBR; 6 General and Colorectal Surgery, Blackpool Teaching Hospitals NHS Foundation Trust, Blackpool, GBR; 7 General Surgery, Craigavon Area Hospital, Northern Ireland Medical and Dental Training Agency (NIMDTA), Portadown, GBR; 8 Pediatric Surgery, Alder Hey Children's Hospital, Liverpool, GBR

**Keywords:** ar, augmented reality, augmented reality (ar), lapar™, surgical education

## Abstract

Augmented reality (AR) is increasingly being integrated into open and laparoscopic surgical education, layering digital guidance onto real-world instruments and tissues to promote safer, more structured skill acquisition. This narrative review synthesizes recent evidence on AR platforms, including video-based overlays, head-mounted displays, and hybrid box-trainer systems, used for basic and advanced tasks, telementoring, and procedural rehearsal. Across surgical specialties, AR-enhanced training consistently improves objective performance metrics, such as task completion times, error rates, and standardized assessment scores (e.g., GOALS/OSATS), compared with conventional methods. It also lowers cognitive workload while enabling standardized feedback and remote expert support. Early integration into robotic surgery training suggests similar benefits in reducing learning curves and enhancing spatial awareness. However, significant barriers persist, including the technical challenge of accurately registering virtual overlays with real anatomy, ergonomic issues and visual fatigue associated with head-mounted displays, and a lack of hardware and software standardization. Furthermore, the evidence base is dominated by small, single-center studies, which limit the generalizability of findings and confirmation of long-term skill transfer to the operating room. Future priorities should focus on developing higher-fidelity haptics, achieving seamless curricular integration, and conducting multicenter randomized trials that track skill retention and patient-centered outcomes. Ultimately, AR serves as a powerful adjunct rather than a replacement for traditional simulation and mentorship, holding substantial potential to broaden access to high-quality surgical training and enhance operator performance.

## Introduction and background

Augmented reality (AR) technologies have emerged as transformative tools in surgical education, particularly in the domains of laparoscopic and open surgery training [1]. These systems are typically integrated with sensorized training environments, primarily using computer vision technologies to track instruments and trainee movements [2-4].

AR platforms, such as the Microsoft HoloLens and LapAR™, provide real-time overlays of anatomical structures, step-by-step guidance, and objective performance metrics, making them valuable for both novice and experienced surgeons. AR video-based systems have become the dominant platform in laparoscopic training, providing real-time visual guidance, procedural information, and anatomical overlays that enhance skill acquisition and reduce training time [5-7].

Traditionally, surgeons learned by apprenticeship under the mantra "see one, do one, teach one," but this paradigm has shifted as work-hour restrictions and ethical imperatives limit learning opportunities on real patients [8-10]. In response, virtual reality (VR) and AR technologies have gained traction as innovative training tools. VR immerses the user in a completely digital environment, whereas AR overlays digital information onto the physical world, thereby combining the benefits of a realistic tactile experience with interactive virtual guidance [6].

The evidence demonstrates that AR-enhanced training significantly improves surgical performance metrics and decreases cognitive workload during complex procedures [11,12]. This review synthesizes current evidence regarding AR implementation in surgical training environments, identifying predominant technologies.

## Review

Scope and evidence of AR in surgical training

AR applications in surgical training are diverse, ranging from projecting anatomical structures or navigational guides onto a patient mannequin during open surgery to overlaying virtual cues on the laparoscopic camera feed during minimally invasive procedures. Early evidence suggests that such AR systems can enhance trainees' understanding of spatial anatomy, increase procedural precision, and improve confidence compared with conventional training alone [11,13].

In orthopedics, AR‐based simulators for procedures such as total hip arthroplasty have shown high-fidelity guidance and automated performance evaluation, improving training efficiency and accuracy [14]. Within urology, head-mounted AR devices (e.g., Google Glass) have been evaluated in nephrectomy, prostatectomy, and implant procedures, with trainees reporting enhanced educational value and feasibility in 31 operations [5]. Finally, in vascular surgery, AR frameworks for endovascular aneurysm repair (EVAR) and vascular access training have been developed, allowing immersive 3D overlays on mannequins or phantoms and demonstrating realistic haptic feedback for simulation [15].

Systematic reviews and meta-analyses consistently demonstrate that AR-based training leads to superior outcomes across key educational domains, yielding measurable gains in technical performance and accuracy, enhanced acquisition and retention of procedural knowledge, and higher levels of trainee confidence when compared with traditional instruction or purely virtual (non-AR) training modalities [12,16-18].

A 2023 systematic review identified dozens of AR training platforms and concluded that AR in surgical education is feasible and effective as an adjunct to traditional training. In particular, head-mounted AR displays (e.g., the Microsoft HoloLens) have shown promising results, yielding improved performance metrics in surgical trainees and earning the highest recommendation among AR systems evaluated [6].

**Figure 1:**
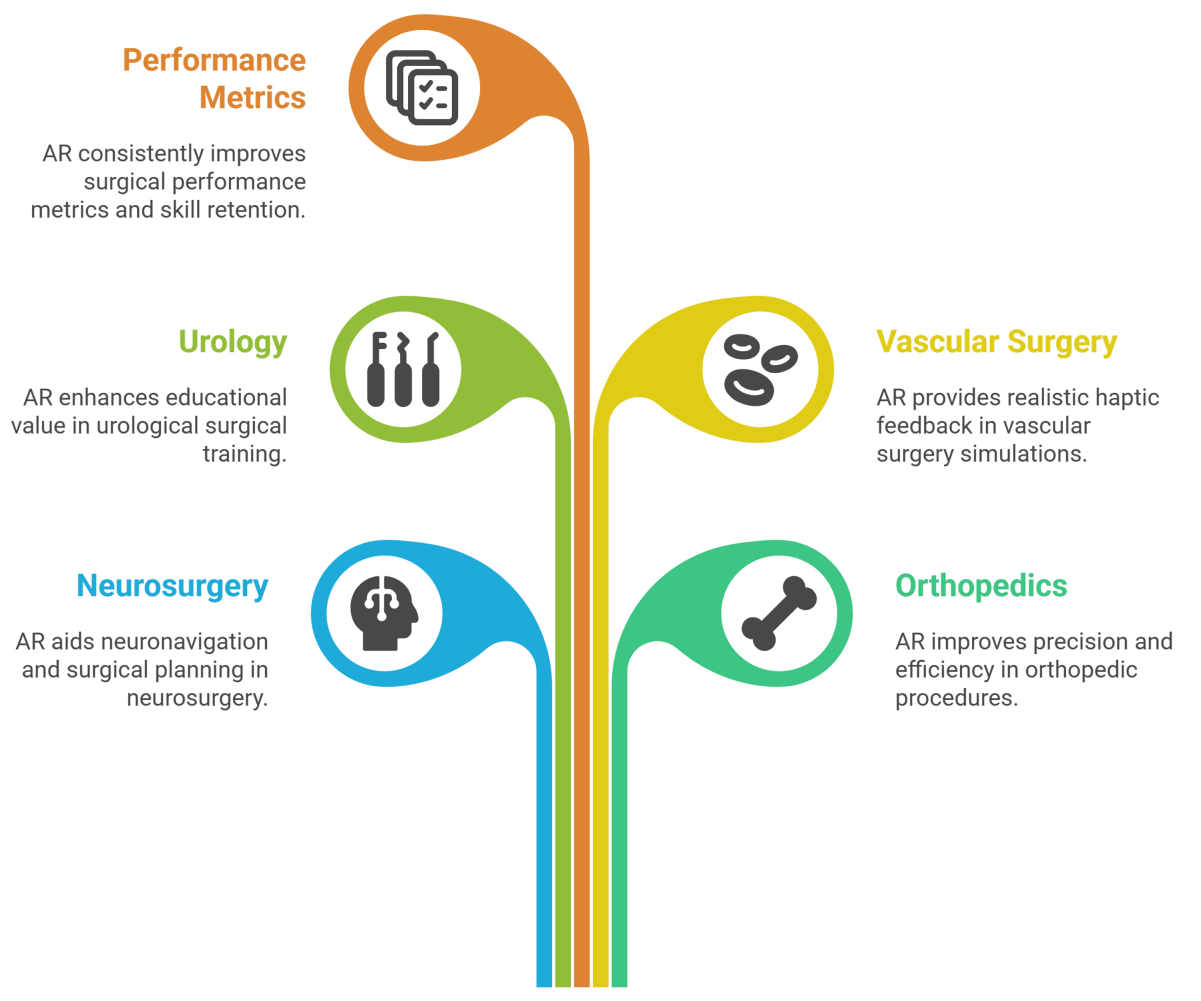
Exploring the use of augmented reality across different specialities Figure credit: Momen Abdelglil Source: [11-19]

Objective performance metrics, most commonly including task or procedure completion time, intraoperative or simulation error rates, and short- and longer-term measures of skill retention, are consistently reported as superior in the AR groups, reflecting faster task execution, fewer mistakes, and more durable maintenance of learned competencies (Figure 1) [19].

AR in open surgical training

Several studies have demonstrated the feasibility of AR systems for training surgeons in open surgery skills. A notable example is an AR suturing self-training system developed by Nagayo et al. [20,21] for teaching medical students how to perform subcuticular skin closure. In this system, expert surgeons first perform the suture on a model while their instrument motions are recorded using motion-capture AR markers. Trainees can then practice on the same model, wearing an AR display that projects a three-dimensional trace of the expert's hands and needle trajectory in real time. This allows novices to observe and imitate the expert's technique from any angle, using actual surgical tools, essentially having a virtual guide for their hand movements. In a randomized controlled trial, the AR suturing trainer was compared to standard video instruction for novice medical students. Both groups showed significant improvements in suturing skill scores after practice. Notably, trainees reported that the motion guidance provided by AR was more helpful for learning instrument handling than watching a video alone. The AR system was rated as equally understandable and easy to use as traditional instructional videos, underscoring that AR can be introduced without diminishing trainee confidence or usability [20,21]. These results suggest that AR-based open surgery trainers can effectively supplement or even replace certain aspects of expert instruction, at least for basic technical skills such as suturing.

Beyond suturing, AR has been applied to more complex open surgical scenarios. In orthopedic surgery, for instance, researchers have explored AR for training procedures such as joint replacement. A systematic review and meta-analysis in 2023 examined extended reality training (including AR and VR) for total hip arthroplasty. They found that trainees who trained with AR achieved more accurate implant positioning (acetabular cup angles closer to ideal) and shorter surgery times in simulations than those who trained by conventional methods [22].

Bui et al. (2025) note that in cardiothoracic surgery, XR technologies enable realistic simulations of rare or complex procedures such as open cardiac bypass or valve repair, giving trainees risk-free practice in managing intricate anatomy and physiology [13]. These AR simulations have been credited with improving trainees' decision-making and procedural confidence in such high-stakes open surgeries. AR is being adopted in oral and maxillofacial surgery for implant placement, orthognathic surgery, and anatomical education [23,24].

AR in laparoscopic surgical training

In an AR-enhanced laparoscopic simulator, the trainee might handle actual laparoscopic instruments and physical objects (providing tactile realism), while a computer overlays digital graphics on the video feed or on a secondary display to provide guidance, performance metrics, or simulated anatomy. One example is the LapAR™ device, a next-generation laparoscopic box trainer incorporating AR that has been evaluated in multicenter studies [25-27].

**Figure 2:**
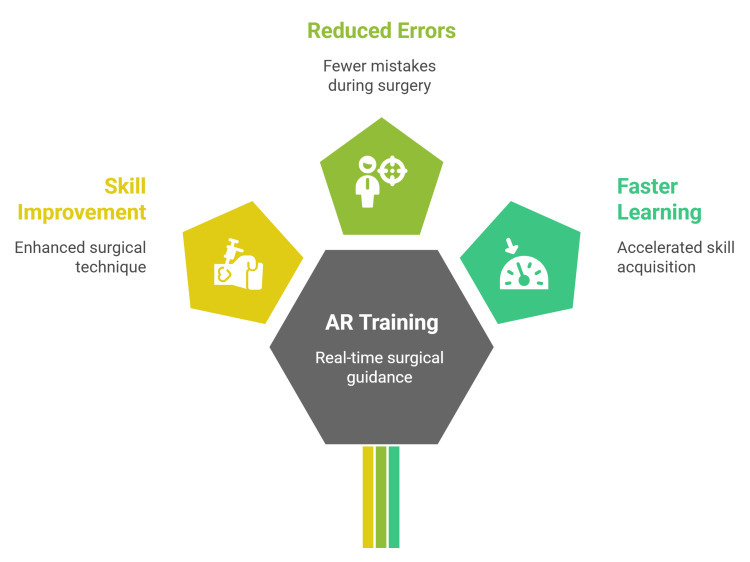
The value of integrating augmented reality into surgical training Figure credit: Momen Abdelglil Source: [20-28]

Real-time AR annotation for surgical education has been shown to improve efficiency and error reduction. In a single-center randomized controlled trial, dynamic AR cues (hand gestures, pointers, and virtual tool overlays) were superimposed on standard laparoscopic views during simulated cholecystectomy tasks. Compared with audio cues and in-person guidance, the AR group completed tasks faster with fewer errors and reported lower cognitive workload, demonstrating that AR can provide an intuitive, standardized feedback mechanism for trainees (Figure 2) [28].

A systematic review and meta-analysis of 12 studies (434 participants) comparing AR-based basic laparoscopic skills training with conventional methods found that AR significantly improved both Operative Assessment of Laparoscopic Skills (GOALS) scores and Objective Structured Assessment of Technical Skills (OSATS) scores (mean differences of 2.40 and 7.71 points, respectively; p<0.001) and reduced subjective workload, underscoring AR's role in speeding skill acquisition and enhancing learner independence [11].

Innovations in AR hardware and software have given rise to specialized simulators designed to replicate laparoscopic procedures with high fidelity. The LapAR™ system, a multi-center pilot device, combines headset-integrated holographic overlays with instrument-tracking sensors to simulate appendectomy tasks under realistic conditions. Trainees using LapAR™ demonstrated marked improvements in completion time, instrument path efficiency, and motion smoothness when compared with consultant benchmarks. Qualitative feedback further highlighted the device's usability and immersive experience, suggesting that portable AR simulators could facilitate distributed learning beyond centralized simulation labs. Such advancements illustrate AR's versatility in teaching both basic skills and complex procedural workflows, emphasizing its role in modern surgical education [7].

AR in robotic surgery

AR is expanding into robotic-assisted surgery. With platforms like da Vinci, AR overlays data on the console or head-mounted displays, providing real-time cues and anatomy highlights. In urologic and neurosurgical cases, overlays project tumor margins and landmarks to aid navigation, turning trainees from observers to guided participants. Early reports suggest faster skill acquisition and shorter learning curves overall, yet dedicated AR robotic training applications and rigorous trials are still limited [29].

Mixed-reality telementoring systems have been developed that enable an expert to annotate a trainee's stereoscopic surgical view and provide remote guidance. In one recent study, a mixed-reality tool enabled experienced surgeons to record robotic procedures and overlay virtual annotations and pointers in 3D for trainees viewing the surgery in a stereoscopic display. In a pilot with 15 surgeons, participants found the system highly useful for robotic surgery training [30].

Pisla et al. (2025) introduced an AR-based robotic surgery trainer for minimally invasive pancreatic surgery that integrates a custom parallel robotic device with the Microsoft HoloLens 2, providing holographic overlays of patient anatomy alongside real-time haptic feedback. Such platforms allow trainees to practice robotic techniques with enhanced spatial awareness. The AR overlays guide instrument positioning and display predicted forces on tissues, thereby enriching the training experience with realistic visual and tactile cues [31]. Early evidence from systematic reviews suggests that extended reality simulators (encompassing AR and VR) can significantly improve technical performance in robot-assisted surgery training. A 2025 meta-analysis found that robotic novices trained with extended reality simulations performed significantly faster on surgical tasks than those with no supplemental training and achieved comparable skill metrics to those trained on traditional physical simulators [32].

Limitations and future directions of AR in surgical training

Accurate alignment of virtual and real-world images (registration) remains a major technical hurdle, especially in anatomically complex or mobile regions. Manual registration is time-consuming and error-prone, while automatic methods are not yet fully reliable [19,33,34]. While head-mounted displays and AR devices are advancing, software integration for seamless, reliable, and useful clinical applications is still lacking. Volatility in the hardware industry also slows progress [1,35]. Users report higher physical discomfort, lower self-perceived performance, and symptoms consistent with visual fatigue when using optical see-through HMDs during task execution, especially with dense overlays [36].

**Figure 3:**
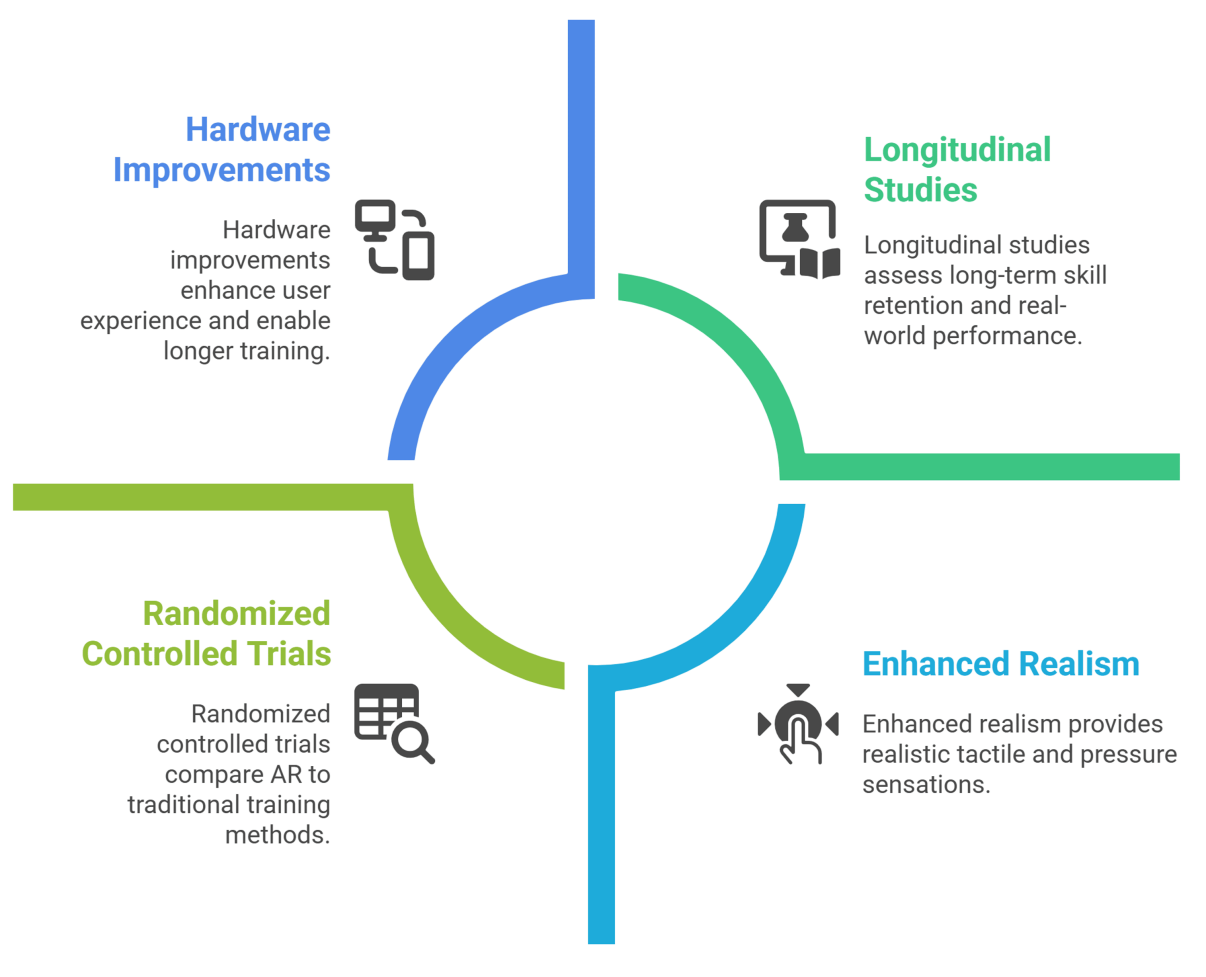
Future directions for augmented reality integration Figure credit: Momen Abdelglil Source: [35-37]

Future research in surgical XR should enhance simulation realism, especially tactile and pressure cues, by advancing haptic feedback and resolving AR hardware limits such as battery life and overheating. Equally, the field needs longitudinal studies and randomized controlled trials to test whether early skill gains persist, transfer to real operations, and improve clinical outcomes. Such trials should track long-term skill retention and patient-relevant metrics (e.g., operating time, complication rates) to establish the sustained effectiveness of VR/AR training beyond the short-term evidence that currently dominates (Figure 3) [37].

## Conclusions

AR has emerged as a powerful adjunct to traditional surgical training, offering enhanced anatomical visualization, real-time procedural guidance, and objective performance feedback that collectively accelerate skill acquisition and reduce errors. Across multiple surgical domains, including open, laparoscopic, and robotic surgery, AR platforms consistently demonstrate superior outcomes compared with conventional training methods, yielding improved task completion times, enhanced instrument-handling accuracy, and reduced cognitive workload. The technology's capacity to provide standardized feedback, enable remote expert mentorship, and democratize access to high-fidelity training experiences positions AR as a valuable educational tool that complements rather than replaces traditional surgical apprenticeship models.
